# The association between lactate and muscle aerobic substrate oxidation: Is lactate an early marker for metabolic disease in healthy subjects?

**DOI:** 10.14814/phy2.14729

**Published:** 2021-02-01

**Authors:** Nicholas T. Broskey, Walter J. Pories, Terry E. Jones, Charles J. Tanner, Donghai Zheng, Ronald N. Cortright, Zhen W. Yang, Nkaujyi Khang, Josh Yang, Joseph A. Houmard, G. Lynis Dohm

**Affiliations:** ^1^ Department of Kinesiology East Carolina University Greenville North Carolina USA; ^2^ Department of Human Performance Laboratory East Carolina University Greenville North Carolina USA; ^3^ Department of Diabetes Obesity Institute East Carolina University Greenville North Carolina USA; ^4^ Department of Surgery East Carolina University Greenville North Carolina USA; ^5^ Department of Physical Therapy East Carolina University Greenville North Carolina USA; ^6^ Department of Physiology East Carolina University Greenville North Carolina USA

**Keywords:** aerobic fitness, lactate, metabolism, mitochondria, skeletal muscle

## Abstract

Fasting plasma lactate concentrations are elevated in individuals with metabolic disease. The aim of this study was to determine if the variance in fasting lactate concentrations were associated with factors linked with cardiometabolic health even in a young, lean cohort. Young (age 22 ± 0.5; *N* = 30) lean (BMI (22.4 ± 0.4 kg/m^2^) women were assessed for waist‐to‐hip ratio, aerobic capacity (VO_2_peak), skeletal muscle oxidative capacity (near infrared spectroscopy; fat oxidation from muscle biopsies), and fasting glucose and insulin (HOMA‐IR). Subjects had a mean fasting lactate of 0.9 ± 0.1 mmol/L. The rate of deoxygenation of hemoglobin/myoglobin (*R* ^2^ = .23, *p* = .03) in resting muscle and skeletal muscle homogenate fatty acid oxidation (*R* ^2^ = .72, *p* = .004) were inversely associated with fasting lactate. Likewise, cardiorespiratory fitness (time to exhaustion during the VO_2_peak test) was inversely associated with lactate (*R* ^2^ = .20, *p* = .05). Lactate concentration was inversely correlated with HDL:LDL (*R* ^2^ = .57, *p* = .02) and positively correlated with the waist to hip ratio (*R* ^2^ = .52, *p* = .02). Plasma lactate was associated with various indices of cardiometabolic health. Thus, early determination of fasting lactate concentration could become a common biomarker used for identifying individuals at early risk for metabolic diseases.

## Graphical abstract

In healthy subjects categorized as normal BMI, lactate was inversely related to skeletal muscle substrate oxidation. Additionally, lactate positively related to markers associated with metabolic disease risks. Lactate may be used as a clinical biomarker to identify patients at early risk for obesity.

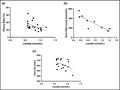

## 1 INTRODUCTION

Elevated fasting plasma lactate concentrations are evident in individuals with metabolic diseases such as obesity, insulin resistance, type 2 diabetes, hypertension, cardiovascular disease, and dyslipidemia (Andersen et al., 2013; Crawford et al., 2010; Lazzeri et al., 2015; Lovejoy et al., 1992; Watanabe et al., 1995). We have previously shown that lactate is indeed elevated in individuals with obesity and was subsequently reduced after 6‐months of endurance training concomitant with reductions in the risk for cardiovascular and metabolic disease (Jones et al., 2019). Weight loss has also been demonstrated to decrease fasting lactate concentrations and improve metabolic health (Chondronikola et al., 2018). Additionally, in a large cohort it was shown that lactate was the most powerful predictor of incident type 2 diabetes (T2D) (Juraschek et al., 2015). The same group conducted a follow‐up study over a 12‐year period in 8,045 subjects and confirmed that elevated fasting lactate preceded type 2 diabetes (Juraschek et al., 2013). All of these studies have led to the conclusion that elevated plasma lactate can serve heath care providers as an early biomarker for predicting metabolic disease. However, the variance in fasting lactate concentrations and relationship to risk factors in a relatively healthy cohort has not been determined.

In terms of risk factors, alterations in skeletal muscle substrate utilization are apparent in individuals with metabolic diseases. We have demonstrated that the capacity for substrate oxidation is depressed in the skeletal muscle of individuals with severe obesity (Houmard et al., 2011). Similarly, decrements in mitochondrial function have been reported in the skeletal muscle of individuals with T2D (Kelley et al., 2002). A deficit in muscle oxidative capacity may also be predictive of future disease in other groups, as children born from mothers with severe obesity preferentially oxidized carbohydrate rather than fat during submaximal exercise (Eaves et al., 2012), while the skeletal muscle of offspring of individuals with T2D display impaired mitochondrial substrate utilization (Befroy et al., 2007). We have reported that primary myotubes from individuals with severe obesity displayed a depressed ability for fat and glucose oxidation which partitioned glucose toward glycolytic end products such as lactate (Houmard et al., 2011; Zou et al., 2018). However, it is not evident if a relationship between indices of muscle oxidative capacity and fasting lactate exist, which may in‐turn provide a mechanistic basis for the predictive ability of lactate concentration and metabolic disease(s). Thus, the purpose of this study was to determine if fasting plasma lactate was related to indicators of muscle oxidative capacity and other indices of cardiometabolic health in a young, healthy cohort and hence, establish a mechanistic foundation for early elevations in plasma lactate preceding metabolic disease(s).

## 2 METHODS

### 2.1 Subjects

Qualified participants reported to the Human Performance Lab building at East Carolina University (ECU). The study was approved by the Institutional Review Board at ECU and all participants provided written informed consent before participation.

Subjects were all non‐pregnant women who classified as non‐smokers and had no known cardiovascular or metabolic diseases. One subset of the subjects (*n* = 21) was tested for VO_2_peak and resting muscle oxygen consumption by near infrared spectroscopy. A second subset of subjects (*n* = 10) had measurement of waist and hip circumference, a muscle biopsy (all but one subject) for measurement of fatty acid oxidation and venipuncture with analysis of plasma lipids.

### 2.2 Blood metabolites

Prior to the visit, subjects fasted, which consisted of refraining from any, food, stimulants (caffeine, nicotine, stimulant medication, etc.), and alcohol for at least 10–12 hr. Subjects also refrained from exercise for 3 days prior. Weight (kg) and height (m) were obtained to calculate BMI (kg/m^2^). A venous blood sample was obtained and subsequently analyzed (Beckman‐Coulter) for fasting glucose, insulin, lactate, and blood lipids.

### 2.3 Cardiorespiratory fitness

Maximal peak oxygen consumption (VO_2_ peak) was determined with a graded cycle exercise protocol to voluntary exhaustion. Oxygen consumption was determined with the True One 2400 metabolic cart (Parvo Medics) and workload increased in 3 min stages. At the end of each stage, a rating of perceived exertion (RPE; Borg Scale) and heart rate (Polar Electro) were recorded. Subjects progressed until maximum effort was achieved or revolutions fell to <40 rpm. All subjects obtained at least 2 of the following criteria: (1) respiratory exchange ratio of 1.10, (2) RPE above 17, and (3) peak heart rate within ±5% of max heart rate. Time to exhaustion was reported as the total time from the start of the exercise test until the subject reached maximal effort.

### 2.4 Near infrared spectroscopy (NIRS)

NIRS is a non‐invasive method of assessing in vivo mitochondrial respiratory capacity and was measured as described previously (Ryan et al., 2014). In short, a cuff around the upper leg was inflated and deoxygenation of hemoglobin/myoglobin in the quadriceps was determined. Subsequently, isometric contraction of the quadriceps was performed for 15–20 s, followed by inflation of the cuff (300 mm Hg) for 5 min to determine total deoxygenated hemoglobin. Percent‐deoxygenated hemoglobin was then calculated by taking rate of deoxygenated hemoglobin divided by the total deoxygenated hemoglobin (µmol/min).

### 2.5 Muscle fatty acid oxidation

Skeletal muscle samples were acquired from percutaneous needle biopsies of the *vastus lateralis* muscle as described (Evans et al., 1982). Subjects were studied between 07:00 and 08:00 after a 12‐hr overnight fast. Subjects were instructed to consume their usual diet and perform normal daily activities for 3 days before the biopsy. Muscle fatty acid (^14^C‐labeled palmitate) oxidation rate was determined in the fresh muscle homogenate as described (Kim et al., 2000).

### 2.6 Statistical analysis

Data were tested for normality via the Shapiro–Wilks test. Pearson product‐moment correlations were used to measure the strength of the association between the variables of plasma lactate and metabolic parameters. Statistical significance was determined as *p* ≤ .05. Statistical analyses were performed using Prism 4.0 software (GraphPad Software, Inc.). Multiple regression analyses were performed using JMP^®^, Version 14 (SAS Institute Inc.).

## 3 RESULTS

All women were young (23 ± 0.5 years) and of normal BMI (22.4 ± 0.4 kg/m^2^). Average fasting plasma lactate was 0.9 ± 0.1 mmol/L. The cohort was also insulin sensitive as classified by HOMA‐IR (1.1 ± 0.1) with fasting glucose values of 88.1 ± 1.0 mg/dL and insulin 4.8 ± 0.4 µIU/ml. Blood cholesterol (194.2 ± 14.9 mg/dl), HDL (68.4 ± 5.8 mg/dl), and triglycerides (89.9 ± 14.6 mg/dl) were all in the normal ranges. Mean LDL was somewhat elevated with an average of 107.9 ± 13.0 mg/dl. Relative VO_2_ peak was 30.2 ± 1.0 ml/kg/min.

**FIGURE 1:**
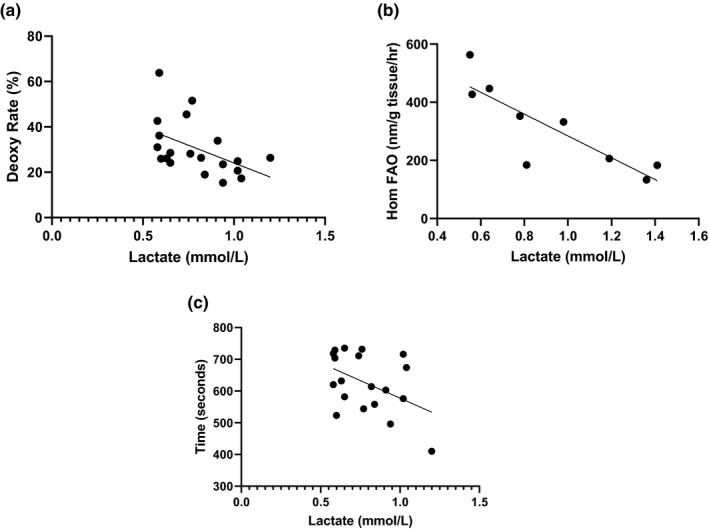
Correlations between skeletal muscle oxidative capacity and fasting plasma lactate. Panel a: Rate of deoxygenated hemoglobin as measured by near infrared spectroscopy. Percent‐deoxy Hb (deoxy rate %) was calculated by taking rate of deoxy Hb dividing the total deoxy Hb (µmol/min). Panel b: Skeletal muscle fatty acid oxidation rate in muscle homogenates acquired via vastus lateralis biopsy. Panel c: Time in seconds to exhaustion during a peak aerobic capacity treadmill test

**FIGURE 2:**
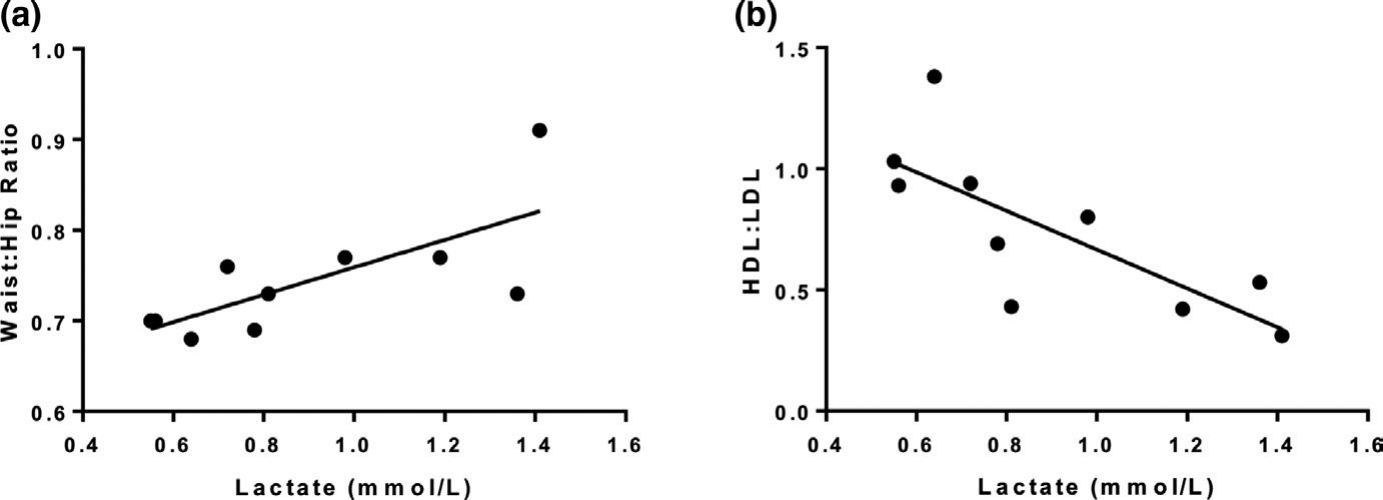
Correlations between parameters associated with metabolic syndrome and fasting plasma lactate

The rates of resting muscle deoxygenation of hemoglobin/myoglobin (*R* ^2 ^= .23, *p* = .03) and skeletal muscle fatty acid oxidation (*R* ^2^ = .72, *p* = .004) were inversely associated with fasting lactate (Figure 1, Panels a and b). Time till exhaustion during the VO_2_ peak test (Figure 1, Panel c) was inversely associated with lactate (*R* ^2^ = .20, *p* = .05). Lactate was positively correlated with waist:hip ratio (*R* ^2^ = .52, *p* = .02) and inversely correlated with HDL:LDL (*R* ^2^ = .57, *p* = .02) (Figure 2 Panels a and b). There were no statistically significant correlations of lactate with the other variables obtained (VO_2_peak: *R* ^2^ = .06, *p* = .31, BMI: *R* ^2^ = .0002, *p* = .98, fasting glucose: *R* ^2^ = .07, *p* = .15, fasting insulin: *R* ^2^ = .08, *p* = .13, HOMA‐IR: *R* ^2^ = .09, *p* = 0.11, data not shown). Lactate was regressed in a model with parameters of muscle aerobic capacity including time to exhaustion, the rate of deoxygenated hemoglobin, and VO_2_ peak. This model was significant (*p* = .02) and explained approximately 46% of the variance in fasting lactate. Time to exhaustion and the rate of deoxygenated hemoglobin were both significant in the model (both *p* = .02); however, VO_2_ peak was not (*p* = .40).

## 4 DISCUSSION

Elevated fasting plasma lactate concentrations are evident in cardiometabolic disease (Andersen et al., 2013; Crawford et al., 2010; Jones et al., 2019; Lazzeri et al., 2015; Lovejoy et al., 1992; Watanabe et al., 1995). Herein, we show that fasting plasma lactate concentration is related to various indices of cardiometabolic health such that those individuals with higher concentrations of lactate display a lower mitochondrial capacity and fatty acid oxidation in skeletal muscle.

The intriguing finding of this study was the inverse relationship between indices of muscle oxidative capacity and fasting lactate. The relationship between NIRS and lactate was of particular interest as it has been shown that NIRS can serve as an in vivo marker of mitochondrial capacity (Ryan et al., 2014). Recently, NIRS has been shown to correlate with aerobic fitness (Beever et al., 2020) although we saw no relationship with VO_2_ peak and lactate in our cohort. However, exercise time to exhaustion was negatively related with fasting plasma lactate concentration and was significant in our regression model. This coupled with a lower *in vivo* oxidative capacity may place these individuals at risk for subsequent metabolic disease. The capacity for mitochondrial substrate oxidation is depressed in the skeletal muscle of subjects with metabolic disease (Houmard et al., 2011; Kelley et al., 2002). These deficits may also be predictive, as children born from mothers with severe obesity preferentially oxidized carbohydrate rather than fat during submaximal exercise (Eaves et al., 2012), while skeletal muscle of offspring of individuals with T2D have lower mitochondrial capacity (Petersen et al., 2004) and impaired substrate utilization (Befroy et al., 2007) compared to those offspring born from parents without. This is particularly relevant to the findings in the present study as these individuals are otherwise young and healthy with no underlying metabolic impairments that would prelude them to metabolic disease yet, similar to our cohort.

There was also an inverse relationship between skeletal muscle fatty acid oxidation and elevated lactate. Fatty acid oxidation was depressed with a compensatory increase in glycolysis in muscle from subjects with severe obesity (Friedman et al., 1994). Lactate can be transported between muscle and liver to make glucose through the Cori Cycle. If this process is accentuated at rest, elevations in fasting blood glucose will occur that may elude to increased risk of glucose intolerance and eventually insulin resistance. We have termed this physiological phenomenon as the “Vicious Cori Cycle”, which could be responsible for elevated glucose in the fasted condition that leads to metabolic disease (Broskey et al., 2020; Pories & Dohm, 2012). The notion of the Vicious Cori Cycle may stem from an initial lesion in the TCA cycle as described in detail elsewhere (Broskey et al., 2020). This is noted in the presence of a low mitochondrial oxidative capacity, which has been associated with dysfunction of the TCA Cycle observed in the obese state (Boyle et al., 2012; Holloway et al., 2009; Houmard et al., 2011; Kelley et al., 1993). Evidence suggests that the TCA Cycle has been mechanistically altered, which results in a shift from oxidative utilization away from carbohydrates and lipids/fatty acids toward glycolysis (Boyle et al., 2012; Kelley et al., 1993). Thus, these current data along with our previous work suggest that the elevated lactate may be due to impairments in whole‐body aerobic capacity and skeletal muscle oxidative capacity as one proposed mechanism.

Our study is not without limitations. The cohort that we studied consists of all women and these results may not be applicable to men. We did not collect data on the timing of the menstrual cycle of these women. However, we do have data from the nine women showing that fasting lactate is stable over the entire period of the menstrual cycle (data not shown). We saw no association between HOMA‐IR and lactate in our cohort, which may prelude that glucose tolerance and/or insulin resistance is disassociated with elevated lactate at this stage in young healthy individuals. Individuals with larger lactate variations (e.g. overweight BMI) prior to developing metabolic diseases could help further disentangle these other relationships. Finally, we acknowledge that our sample size is small regarding skeletal muscle outcomes. However, the relationship with lactate and fatty acid oxidation is strong and the use of the powerful in vivo NIRS technique bolsters these muscle outcomes.

To conclude, this study supports the possibility that fasting lactate could be utilized as a relatively easily obtained and inexpensive biomarker for identifying healthy individuals at early risk for developing metabolic disease(s). Blood lactate concentrations could be the first step of “staging” metabolic syndrome. This is in anticipation of providing a similar staging system such as that used in cancer, which would ultimately guide more targeted treatments throughout disease progression.

## CONFLICT OF INTEREST

The authors declared no conflicts of interest.

## AUTHOR CONTRIBUTIONS

N.T.B. wrote the manuscript, researched data, performed statistical analyses. W.J.P. conceived the study, reviewed/edited the manuscript, T.E.J. researched data, reviewed/edited the manuscript, C.J.T. researched data, reviewed/edited the manuscript. D.Z. assisted with experiments, researched data, reviewed the manuscript. R.N.C. reviewed/edited the manuscript, Z.W.Y researched data, reviewed the manuscript. N.K. researched data, performed statistical analyses, reviewed/edited the manuscript. M.S., J.Y. researched data, reviewed/edited the manuscript, J.A.H. conceived the study, researched data, reviewed/edited the manuscript. G.L.D. conceived the study, researched data, reviewed/edited the manuscript.

## References

[phy214729-bib-0001] Andersen, L. W. , Mackenhauer, J. , Roberts, J. C. , Berg, K. M. , Cocchi, M. N. , & Donnino, M. W. (2013). Etiology and therapeutic approach to elevated lactate levels. Mayo Clinic Proceedings, 88, 1127–1140. 24079682 10.1016/j.mayocp.2013.06.012 PMC3975915

[phy214729-bib-0002] Beever, A. T. , Tripp, T. R. , Zhang, J. , & MacInnis, M. J. (2020). NIRS‐derived skeletal muscle oxidative capacity is correlated with aerobic fitness and independent of sex. Journal of Applied Physiology, 129, 558–5568. 32702279 10.1152/japplphysiol.00017.2020 PMC7517427

[phy214729-bib-0003] Befroy, D. E. , Petersen, K. F. , Dufour, S. , Mason, G. F. , de Graaf, R. A. , Rothman, D. L. , & Shulman, G. I. (2007). Impaired mitochondrial substrate oxidation in muscle of insulin‐resistant offspring of type 2 diabetic patients. Diabetes, 56, 1376–1381. 17287462 10.2337/db06-0783 PMC2995532

[phy214729-bib-0004] Boyle, K. E. , Zheng, D. , Anderson, E. J. , Neufer, P. D. , & Houmard, J. A. (2012). Mitochondrial lipid oxidation is impaired in cultured myotubes from obese humans. International Journal of Obesity, 36, 1025–1031. 22024640 10.1038/ijo.2011.201 PMC3848508

[phy214729-bib-0005] Broskey, N. T. , Zou, K. , Dohm, G. L. , & Houmard, J. A. (2020). Plasma lactate as a marker for metabolic health. Exercise and Sport Sciences Reviews, 48, 119–124. 32271180 10.1249/JES.0000000000000220 PMC7311283

[phy214729-bib-0006] Chondronikola, M. , Magkos, F. , Yoshino, J. , Okunade, A. L. , Patterson, B. W. , Muehlbauer, M. J. , Newgard, C. B. , & Klein, S. (2018). Effect of progressive weight loss on lactate metabolism: A randomized controlled trial. Obesity (Silver Spring), 26, 683–688. 29476613 10.1002/oby.22129 PMC5866193

[phy214729-bib-0007] Crawford, S. O. , Hoogeveen, R. C. , Brancati, F. L. , Astor, B. C. , Ballantyne, C. M. , Schmidt, M. I. , & Young, J. H. (2010). Association of blood lactate with type 2 diabetes: the Atherosclerosis Risk in Communities Carotid MRI Study. International Journal of Epidemiology, 39, 1647–1655. 20797988 10.1093/ije/dyq126 PMC2992628

[phy214729-bib-0008] Eaves, A. D. , Colon, A. , Dubose, K. D. , Collier, D. , & Houmard, J. A. (2012). Substrate utilization during submaximal exercise in children with a severely obese parent. Nutrition & Metabolism, 9, 38. 22571243 10.1186/1743-7075-9-38 PMC3422990

[phy214729-bib-0009] Evans, W. J. , Phinney, S. D. , & Young, V. R. (1982). Suction applied to a muscle biopsy maximizes sample size. Medicine and Science in Sports and Exercise, 14, 101–102. 7070249

[phy214729-bib-0010] Friedman, J. E. , Caro, J. F. , Pories, W. J. , Azevedo, J. L. Jr , & Dohm, G. L. (1994). Glucose metabolism in incubated human muscle: effect of obesity and non‐insulin‐dependent diabetes mellitus. Metabolism, 43, 1047–1054. 8052146 10.1016/0026-0495(94)90188-0

[phy214729-bib-0011] Holloway, G. P. , Bonen, A. , & Spriet, L. L. (2009). Regulation of skeletal muscle mitochondrial fatty acid metabolism in lean and obese individuals. The American Journal of Clinical Nutrition, 89, 455S–462S. 19056573 10.3945/ajcn.2008.26717B

[phy214729-bib-0012] Houmard, J. A. , Pories, W. J. , & Dohm, G. L. (2011). Is there a metabolic program in the skeletal muscle of obese individuals? Journal of Obesity, 2011, 250496. 21603262 10.1155/2011/250496 PMC3092539

[phy214729-bib-0013] Jones, T. E. , Pories, W. J. , Houmard, J. A. , Tanner, C. J. , Zheng, D. , Zou, K. , Coen, P. M. , Goodpaster, B. H. , Kraus, W. E. , & Dohm, G. L. (2019). Plasma lactate as a marker of metabolic health: Implications of elevated lactate for impairment of aerobic metabolism in the metabolic syndrome. Surgery, 166, 861–866. 31253418 10.1016/j.surg.2019.04.017 PMC7142375

[phy214729-bib-0014] Juraschek, S. P. , Bower, J. K. , Selvin, E. , Subash Shantha, G. P. , Hoogeveen, R. C. , Ballantyne, C. M. , & Young, J. H. (2015). Plasma lactate and incident hypertension in the atherosclerosis risk in communities study. American Journal of Hypertension, 28, 216–224. 24994607 10.1093/ajh/hpu117 PMC4357800

[phy214729-bib-0015] Juraschek, S. P. , Selvin, E. , Miller, E. R. , Brancati, F. L. , & Young, J. H. (2013). Plasma lactate and diabetes risk in 8045 participants of the atherosclerosis risk in communities study. Annals of Epidemiology, 23, 791–796 e794. 24176820 10.1016/j.annepidem.2013.09.005 PMC4034672

[phy214729-bib-0016] Kelley, D. E. , He, J. , Menshikova, E. V. , & Ritov, V. B. (2002). Dysfunction of mitochondria in human skeletal muscle in type 2 diabetes. Diabetes, 51, 2944–2950. 12351431 10.2337/diabetes.51.10.2944

[phy214729-bib-0017] Kelley, D. E. , Mokan, M. , Simoneau, J. A. , & Mandarino, L. J. (1993). Interaction between glucose and free fatty acid metabolism in human skeletal muscle. The Journal of Clinical Investigation, 92, 91–98. 8326021 10.1172/JCI116603 PMC293539

[phy214729-bib-0018] Kim, J. Y. , Hickner, R. C. , Cortright, R. L. , Dohm, G. L. , & Houmard, J. A. (2000). Lipid oxidation is reduced in obese human skeletal muscle. American Journal of Physiology‐Endocrinology and Metabolism, 279, E1039–E1044. 11052958 10.1152/ajpendo.2000.279.5.E1039

[phy214729-bib-0019] Lazzeri, C. , Valente, S. , Chiostri, M. , & Gensini, G. F. (2015). Clinical significance of lactate in acute cardiac patients. World Journal of Cardiology, 7, 483–489. 26322188 10.4330/wjc.v7.i8.483 PMC4549782

[phy214729-bib-0020] Lovejoy, J. , Newby, F. D. , Gebhart, S. S. , & DiGirolamo, M. (1992). Insulin resistance in obesity is associated with elevated basal lactate levels and diminished lactate appearance following intravenous glucose and insulin. Metabolism, 41, 22–27. 10.1016/0026-0495(92)90185-d 1538640

[phy214729-bib-0021] Petersen, K. F. , Dufour, S. , Befroy, D. , Garcia, R. , & Shulman, G. I. (2004). Impaired mitochondrial activity in the insulin‐resistant offspring of patients with type 2 diabetes. New England Journal of Medicine, 350, 664–671. 10.1056/NEJMoa031314 PMC2995502 14960743

[phy214729-bib-0022] Pories, W. J. , & Dohm, G. L. (2012). Diabetes: have we got it all wrong? Hyperinsulinism as the culprit: surgery provides the evidence. Diabetes Care, 35, 2438–2442. 23173133 10.2337/dc12-0684 PMC3507594

[phy214729-bib-0023] Ryan, T. E. , Brophy, P. , Lin, C. T. , Hickner, R. C. , & Neufer, P. D. (2014). Assessment of in vivo skeletal muscle mitochondrial respiratory capacity in humans by near‐infrared spectroscopy: a comparison with in situ measurements. The Journal of Physiology, 592, 3231–3241. 24951618 10.1113/jphysiol.2014.274456 PMC4146372

[phy214729-bib-0024] Watanabe, R. M. , Lovejoy, J. , Steil, G. M. , DiGirolamo, M. , & Bergman, R. N. (1995). Insulin sensitivity accounts for glucose and lactate kinetics after intravenous glucose injection. Diabetes, 44, 954–962. 7622002 10.2337/diab.44.8.954

[phy214729-bib-0025] Zou, K. , Hinkley, J. M. , Park, S. , Zheng, D. , Jones, T. E. , Pories, W. J. , Hornby, P. J. , Lenhard, J. , Dohm, G. L. , & Houmard, J. A. (2018). Altered tricarboxylic acid cycle flux in primary myotubes from severely obese humans. International Journal of Obesity, 43, 895–905. 29892037 10.1038/s41366-018-0137-7 PMC13492247

